# ﻿Notes on spotted-elytron species of *Gallerucida* Motschulsky with the description of six new species from China (Coleoptera, Chrysomelidae, Galerucinae)

**DOI:** 10.3897/zookeys.1116.85987

**Published:** 2022-08-04

**Authors:** Si-yuan Xu, Rui-E Nie, Xing-ke Yang

**Affiliations:** 1 Key Laboratory of Zoological Systematics and Evolution, Institute of Zoology, Chinese Academy of Sciences, 1 Beichen West Road, Chaoyang District, Beijing 100101, China Institute of Zoology, Chinese Academy of Sciences Beijing China; 2 University of Chinese Academy of Sciences, No. 19(A) Yuquan Road, Shijingshan District, Beijing, 100049, China University of Chinese Academy of Sciences Beijing China; 3 Anhui Provincial Key Laboratory of the Conservation and Exploitation of Biological Resources, College of Life Sciences, Anhui Normal University, Wuhu, 241000, China Anhui Normal University Wuhu China; 4 Guangdong Key Laboratory of Animal Conservation and Resource Utilization, Guangdong Public Laboratory of Wild Animal Conservation and Utilization, Guangdong Institute of Applied Biological Resources, Guangzhou 510260, China Guangdong Institute of Applied Biological Resources Guangzhou China

**Keywords:** Coleoptera, Chrysomelidae, Galerucinae, *Gallerucida*, new species

## Abstract

In this study, fifteen species of *Gallerucida* Motschulsky, 1860 (Coleoptera: Chrysomelidae: Galerucinae), with spotted elytra, from China are reviewed, including one new record: *G.balyi* (Duvivier, 1885), six new species: *G.fortispina* Xu & Yang, **sp. nov.**, *G.levifasciata* Xu & Nie, **sp. nov.**, *G.nigrovittata* Xu & Yang, **sp. nov.**, *G.octodecimpunctata* Xu & Yang, **sp. nov.**, *G.piceusfasciata* Xu & Yang, **sp. nov.**, *G.rufipectoralis* Xu & Nie, **sp. nov.**, and *Aplosonyxgansuica* (Chen, 1942), **comb. nov**. is removed from genus *Gallerucida*. A key to the spotted-elytron species of *Gallerucida* from China is given as well as habitus photographs of the related species and *Aplosonyxgansuica***comb. nov.** and photographs of the aedeagus of each new species.

## ﻿Introduction

*Gallerucida* Motschulsky, with 81 species described so far, is one of the largest genera in Galerucinae (Coleoptera: Chrysomelidae). All known species are distributed in the Palearctic region and Oriental region, of which 64 species are recorded in China (Yang et al. 2015). Larvae and adults feed on leaves, and some species are severe pests of commercial crops.

Due to the rich species diversity of *Gallerucida* in China, in this study we focus on the spotted-elytron species of *Gallerucida* from China, but this group, comprising 14 species, may not be a natural group. We describe six new species and record one new species from China, give habitus and aedeagus images and a key to species from China.

## ﻿Materials and methods

The specimens were examined with an Olympus SZ61 microscope. Abdomens and aedeagus of each species were dissected using the following procedure: for dried or ethanol preserved specimens, the abdomen was separated and transferred to a vial containing 10% KOH which was heated in a boiling water bath for 20–25 min. The abdomen was moved to a clean cavity slide and the abdomen pressed with the back of a dissecting needle to extrude and separate the aedeagus. The abdomen and aedeagus were then washed with distilled water and carefully moved using fine forceps to another cavity slide containing glycerin for examination.

Habitus images were taken using a Canon EOS 5DSR digital camera, equipped with MP-E 65 mm f/2.8 lens. Illumination was by flash, and each photo was taken by a macro slide system.

Aedeagus images were taken using a Nikon D610 digital camera, linking a Zeiss microscope, with 5× objective lens. A cable shutter release was used to prevent the camera from shaking. The depth of field was determined by different sizes of the aedeagus. Helicon Focus 6 (http://www.heliconsoft.com/heliconsoft-products/helicon-focus) stacked full depth of images. Adobe Photoshop CC (https://www.photoshop.com) edited images and resulted output.

The label data were translated into English from the original Chinese. Accurate labelling data for all type specimens of species: A slash (/) divides the date into different lines of a label. A double slash (//) separates the data of different labels. Type specimens of the six new species are deposited in the Institute of Zoology, Chinese Academy of Sciences, Beijing, China (**IZAS**). Abbreviations used in the paper are **TL**: type locality and **TD**: type depository.

Specimens studied herein are deposited at the following institutes and collections:

**ISNB** Institut Royal des Sciences naturelles de Belgique, Bruxelles, Belgium;

**IZAS** Institute of Zoology, Chinese Academy of Sciences, Beijing, China;

**MNHN**Museum national d’Histoire naturelle, Paris, France;

**NHMB**Naturhistorisches Museum, Basel, Switzerland;

**NHMUK**The Natural History Museum, London, UK;

**SDEI**Senckenberg Deutsches Entomologisches Institut, Müncheberg, Germany.

## ﻿Taxonomy

### ﻿Genus Gallerucida

Taxon classificationAnimaliaColeopteraChrysomelidae

Motschulsky

F7EA6781-5264-54DD-AE38-F63ECEA06011

Gallerucida Motschulsky, 1861: 24. Type species: Gallerucidabifasciata Motschulsky, 1861, designated by Weise 1924.
Eustetha Baly, 1861: 296. Type species: Eustethaflaviventris Baly, 1861, by original designation. Synonymized by Chûjô 1962: 147.
Melospila Baly, 1861: 297. Type species: Melospilanigromaculata Baly, 1861, by monotypy and original designation. Synonymized by Chapuis 1875: 227.
Hylaspes Baly, 1865: 436. Type species: Hylaspeslongicornis Baly, 1865, by monotypy and original designation. Synonymized by Gressitt and Kimoto 1963: 717.
Galerucida : Chapuis 1875: 224, 227. error or emendation for Gallerucida Motschulsky, 1861: 24.
Stethidea Baly, 1890: 13. Type species: Doryidabalyi Duvivier, 1885, by monotypy and original designation. Synonymized by Kimoto 1989: 234.
Coptomesa Weise, 1912: 91. Type species: Gallerucida (Coptomesa) maculata Weise, 1912, by monotypy. Synonymized by Kimoto 1965: 398.

#### Distribution.

Palaearctic region, Oriental region.

### ﻿Key to the spotted-elytron species of *Gallerucida* from China

**Table:** 

1	Pronotum black or atropurpureus (dark purple)	**2**
–	Pronotum not as above	**8**
2	Pronotum atropurpureus	**3**
–	Pronotum black	**4**
3	From scutellum to middle area of elytra with an irregular brown band, after middle with 5 small brownish spots, arranged in two rows as 4:1	***G.levifasciata* sp. nov.**
–	Basal area of elytra with one atropurpureus oval spot adjacent to the scutellum, middle area of elytra with a transverse atropurpureus stripe and subapically with an atropurpureus spot	***G.ornatipennis* (Duviver, 1885)**
4	Antennae black	**5**
–	Antennae not black	**6**
5	Elytra yellowish brown, with a round black spot adjacent to the scutellum, middle area of elytra with a transverse black stripe followed by 4 black spots, arranged two rows as 3:1	***G.bifasciata* Motschulsky, 1860**
–	Elytra black, with two oblique transverse yellow stripes	***G.tenuefasciata* Fairmaire, 1888**
6	Ventral surface totally black, elytra with three wide subparallel transverse atropurpureus stripes and a rounded atropurpureus spot near apex	***G.rubrozonata* Fairmaire, 1889**
–	Ventral surface totally brown or only ventral surface of the thorax black	**7**
7	Ventral surface of thorax black, after middle of elytra with a transverse black stripe	***G.nigrovittata* sp. nov.**
–	Ventral surface of thorax and abdomen totally brown	***G* . *nigropicta* Fairmaire, 1888**
8	Pronotum and ventral surface dark green	***G.piceusfasciata* sp. nov.**
–	Pronotum not dark green	**9**
9	Pronotum with black spots	**10**
–	Pronotum without spots	**11**
10	Antennae brown except four basal segments orange	***G.octodecimpunctata* sp. nov.**
–	Antennae yellow	***G.balyi* (Duvivier, 1885)**
11	Pronotum yellow, ventral surface yellowish brown	**12**
–	Pronotum reddish brown, ventral surface black	**13**
12	Head black	***G.fortispina* sp. nov.**
–	Head yellow or reddish brown	***G.sauteri* Chûjô, 1938**
13	Elytra yellow, middle area with a transverse black stripe	***G* . *tricolor* Gressitt & Kimoto, 1963**
–	Elytra yellowish brown, basal area with a slim black stripe, subapically with a wide transverse black stripe	***G* . *rufipectoralis* sp. nov.**

### ﻿ Gallerucida balyi

Taxon classificationAnimaliaColeopteraChrysomelidae

(Duvivier, 1885) (new record)

50A5E911-3D52-5129-950C-2B2DCF3A677F

Fig. 43

Doryida balyi Duvivier, 1885: 394. **TL** Malaysia: Malacca; **TD**ISNB
Stethidea fulva Laboissière, 1931: 135
Stethidea maculata Laboissière, 1931: 133 (nec Galerucidamaculata Weise, 1912)
Stethidea oranta Laboissière, 1932: 132 (nom. nov. for Stethideamaculata Laboissière, nec Weise)
Gallerucida balyi : Kimoto, 1989: 234.

#### Type material.

***Paratype***: labels: V. Laboissière vid., 1939 / *Stethidea* / *Balyi* Duviv. / Type // sec. Weise, Col. Cat. / Junk (78), 1924, p143: / *Stethidea* / *Balyi* Duv. // cf. Stett. Ent. Zeitung. / XlVI, 1885, p.394 // Type // det. Duvivier. / *Doryida* / *balyi* Duviv. // Type // Collect. / Duvivier. (ISNB). ***Syntype***: labels: *Doryida* / *balyi* / Duvivier / halacca. // SYN-TYPE. // 37 // Baly coll. (NHMUK).

#### Other material examined.

1♂, China, Guangxi, Longzhou, Sanlian, 350 m, 13-VI-2000, Wen-Zhu Li, leg. (IZAS).

#### Distribution.

China: Guangxi. India; Myanmar; Thailand; Laos; Vietnam; Malaysia: Malacca.

### ﻿ Gallerucida bifasciata

Taxon classificationAnimaliaColeopteraChrysomelidae

Motschulsky, 1861

336F4FDA-7776-5BCF-81C2-97678BBDFB8F

Fig. 44

Gallerucida bifasciata Motschulsky, 1861: 24. **TL** Japan; **TD**MNHN.
Melospila nigromaculata Baly, 1861: 297. Synonymized by Kimoto 1965: 399.
Melospila consociata Baly, 1874: 184. Synonymized by Ogloblin 1936: 354.
Galerucida nigrofasciata Baly, 1879: 453. Synonymized by Gressitt and Kimoto 1963: 722.
Galerucida nigrita Chûjô, 1935: 168. Synonymized by Kimoto 1966: 34.
Galerucida bifasciata nigromaculata Takizawa, 1980: 73. Synonymized by Takizawa 1985: 10.

#### Type material.

***Syntype***: labels: *Galerucida* / *bifasciata* / Motch. / Type / Japonia // Ex-Musaeo / E. Harold. (MNHN).

#### Other material examined

**(115 spec.).** China: **Heilongjiang**: 1♀, Heilongjiang, Jingpo Hu, 6-IX-1970, unknown, leg.; 3♂♂, Heilongjiang, Harbin Shi, (20-22)-VI-1955, unknown, leg.; **Jinlin**: 2♀♀, 1♂, Jilin, Linjiang, 14-V-1955, unknown, leg.; **Liaoning**: 1♂, Liaoning, Qianshan, 15-V-1955, unknown, leg.; 1♂, Liaoning, Qingyuan, 3-VI-1955, unknown, leg.; **Gansu**: 3♀♀, 5♂♂, Gansu, Huixian, Yuguan, 23-V-1981, unknown, leg.; **Hebei**: 2♂♂, Hebei, Wuling Mountain, Shizigou [Zishigou], 500 m, 2-VI-1981, Pei-Yu Yu, leg.; 1♂, Hebei, Yu Xian, Longquanguan, 3-VIII-1998, Ai-Min Shi et al., leg.; 1♂, Hebei, Wuling Mountain, 1200–1800 m, 5-VII-1963, Sheng-Qiao Jiang, leg.; **Shaanxi**: 2♀♀, 1♂, Shaanxi, Hua Mountain, 9-VI-1936, unknown, leg.; 1♂, 1♀, Shaanxi, Hua Mountain, 1670 m, 29-V-1963, Jin-Long Mao, leg.; 1♂, Shaanxi, Hua Mountain, 1000 m, 10-VII-1972, Shu-Yong Wang, leg.; 1♀,1♂, Shaanxi, Ningxia Xian, Shibazhang, 1150 m, 28-VI-1999, De-Cheng Yuan, leg.; **Henan**: 1♀, Henan, Luanchuan, Longyuwan, 1000–1400 m, 12-VII-1996, Wan-Zhi Cai, leg.; 2♀♀, Henan, Jigong Mountain, 18-VII-1960, unknown, leg.; 1♂, Henan, Jigong Mountain, 8-VI-1982, unknown, leg.; 1♀, Henan, Jigong Mountain, 8-VI-1982, unknown, leg.; 1♂, Henan, Luoyang, 18-VI-1936, unknown, leg.; **Jiangsu**: 2♂♂, Jiangsu, Nanjing, 6-V-1936, unknown, leg.; 1♂, Jiangsu, Nanjing, 22-VI-1923, unknown, leg.; **Anhui**: 1♀, Anhui, Jinzhai Xian, Baojiawo, Linchang, 524.5 m, 1-VIII-2021, Zheng-Yu Zhao, leg.; **Zhejiang**: 1♀, Zhejiang, Mogan Mountain, 8-V-1935, unknown, leg.; 3♂♂, Zhejiang, Tianmu Mountain, 300–900 m, 24-VI-1957, unknown, leg.; 1♀, Zhejiang, Anji Xian, Longwang Mountain, 450 m, 16-V-1996, Hong Wu, leg.; 1♀, Zhejiang, Anji Xian, Longwang Mountain, 500 m, 17-V-1996, Hong Wu, leg.; 1♀, Zhejiang, Anji Xian, Longwang Mountain, 500 m, 12-VI-1996, Xing-Ke Yang, leg.; 1♂, Zhejiang, Hangzhou Shi, 12-VI-1954, unknown, leg.; **Hubei**: 1♂, Hubei, Badong, Sanxia, Linchang, 180 m, 27-VI-1993, Wen-Zhu Li, leg.; 2♀♀, Hubei, Zigui Xian, Maoping, 80 m, 28-IV-1994, Jian Yao, leg.; 1♀, Hubei, Zigui Xian, Jiulingtou, 110 m, 1-V-1994, You-Wei Zhang, leg.; 1♀, Hubei, Xingshan, Xiakou, 140 m, 2-V-1994, You-Wei Zhang, leg.; 1♂, Hubei, Badong, Dongxiangkou, 100 m, 14-V-1994, Wen-Zhu Li, leg.; 1♂, Hubei, Zigui Xian, Maoping, 170 m, 28-IV-1994, Wen-Zhu Li, leg.; 7♀♀, 1♂, Hubei, Zigui Xian, Jiulingtou, 100 m, 13-VI-1993, Wen-Zhu Li, leg.; **Jiangxi**: 1♀, Jiangxi, Yishouyuanqian, 9-V-1959, unknown, leg.; **Hunan**: 1♂, Hunan, Yongshun, Shanmuhe, Linchang, 500 m, 7-VIII-1988, Liu Hong, leg.; 1♀, Hunan, Yongshun, Shanmuhe, Linchang, 600 m, 6-VIII-1988, Shu-Yong Wang, leg.; 1♂, Hunan, Nanyue, 1300 m, VII-1963, unknown, leg.; 1♀, Hunan, Zhangjiajie, 16-VII-1988, Yong-Kun Li, leg.; **Fujian**: 1♀, Fujian, Jianyang, Huangkeng, 290–320 m, 2-IV-1960, Fu-Ji Pu, leg.; 1♀, Fujian, Jianyang, Huangkeng, 290–320 m, 6-IV-1960, Fu-Ji Pu, leg.; 1♀, Fujian, Jianyang, Huangkeng, 290 m, 27-III-1960, Sheng-Qiao Jiang, leg.; 1♀, Fujian, Jianyang, Huangkeng, Guilin, 290–400 m, 5-IV-1960, Sheng-Qiao Jiang, leg.; 1♀, Fujian, Jianyang, Huangkeng, Guilin, 290–400 m, 5-IV-1960, Sheng-Qiao Jiang, leg.; 1♀, Fujian, Jianyang, Huangkeng, Guilin, 290 m, 10-IV-1960, Sheng-Qiao Jiang, leg.; 1♂, Fujian, Jianyang, Huangkeng, 290–320 m, 6-IV-1960, Fu-Ji Pu, leg.; 1♂, Fujian, Jianyang, Huangkeng, 290 m, 2-IV-1960, Fu-Ji Pu, leg.; 1♂, Fujian, Jianyang, Huangkeng, Aotou, 950 m, 3-VI-1960, Sheng-Qiao Jiang, leg.; 1♂, Fujian, Jianyang, Huangkeng, Guilin, 290–320 m, 14-IV-1960, Fu-Ji Pu leg.; 1♀, 2♂♂, Fujian, Jianyang, Huangkeng, Guilin, 290 m, 11-IV-1960, Sheng-Qiao Jiang, leg.; **Guangxi**: 2♀♀, 1♂, Guangxi, Guilin, 150 m, 20-V-1963, Yong-Shan Shi, leg.; 1♀, 2♂♂, Guangxi, Longsheng, Sanmen, 300 m, 31-V-1963, leg.; 5♀♀, 6♂♂, Guangxi, Guilin, 150 m, 20-V-1963, Yong-Shan Shi, leg.; 2♀♀, Guangxi, Luoxiang, 200 m, 15-V-1985, Xue-Zhong Zhang, leg.; **Sichuan**: 1♀, Sichuan, Emei Mountain, Baoguosi, 3-VI-1957, Ke-Ren Huang, leg.; 1♀, Sichuan, Emei Mountain, 580 m, 24-VI-1955, Tian-rong Huang, leg.; 1♂, Sichuan, Emei Mountain, 580–650 m, 20-VI-1955, Bing-rong Ou, leg.; 1♀, 4♂♂, Sichuan, Emei Mountain, 580 m, 24-VI-1955, Tian-rong Huang, leg.; 2♂♂, Sichuan, Guan Xian, 700–1000 m, 29-IV-1963, Xue-Zhong Zhang, leg.; 1♂, Sichuan, Xichang, Zhaojue, 2100 m, VI-1998, Rong-Hua Guo, leg.; **Guizhou**: 1♀, Guizhou, Leishan, Taojiang, 1000 m, 5-VII-1988, Long-Long Yang, leg.; 1♂, Guizhou, Leishan, Taojiang, 950 m, 7-VII-1988, Xing-Ke Yang, leg.; 1♀, Guizhou, Huaxi, 20-IV-1948, unknown, leg.; 1♀, Guizhou, Maolan, Xiaoqikong, 30-V-1998, Liang-Zhang Song, leg.; **Yunnan**: 1♂, Yunnan, Zhenxiong Xian, 1850 m, Jia-Hua Zhen, 11-V-1980, leg. (all IZAS).

#### Distribution.

China: Heilongjiang, Jilin, Liaoning, Gansu, Hebei, Shaanxi, Henan, Jiangsu, Anhui, Zhejiang, Hubei, Jiangxi, Hunan, Fujian, Taiwan, Guangxi, Sichuan, Guizhou, Yunnan.

#### Host plants.

*Reynoutriajaponica*, *Fagopyrumesculentum*, *Rumexacetosa*, *Polygonum* sp., *Prunuspersica*, *Rheumofficinale*, *Polygonummultiflorum*, *Spiraeasalicifolia*.

### ﻿ Gallerucida nigropicta

Taxon classificationAnimaliaColeopteraChrysomelidae

Fairmaire, 1888

3E2C4EE2-2BD9-57C0-AE1D-770EDC1A1402

Fig. 45

Galerucida nigropicta Fairmaire, 1888: 40. **TL** China: Yunnan; **TD**MNHN.
Eustetha nigropuncta Fairmaire, 1889: 79. Synonymized by Gressitt and Kimoto 1963: 727.
Galerucida nigropicta fulvicollis Laboissière, 1934: 119. Synonymized by Gressitt and Kimoto 1963: 727.
Galerucida nigropuncta : Ogloblin 1936: 362, 442.
Gallerucida nigropicta : Gressitt and Kimoto 1963: 727.

#### Type material.

***Syntypes***: labels: China, Yunnan // *Galerucida* / *nigropicta* / Fairm. // Ex-Musaeo / L. Fairmaire / 1893. (MNHN).; China, Yunnan // Ex-Musaeo / L. Fairmaire / 1893. (MNHN).

#### Other material examined

**(2 spec.)**. China: **Guizhou**: 1♂, Guizhou, Guiyang, VI-VII-1981; **Yunnan**: 1♂, Yunnan, Yanjin, Lijiang, Yulong Xueshan, 14-VIII-1979, Ling zuo-pei, leg. (all IZAS).

#### Distribution.

China: Gansu, Hubei, Sichuan, Guizhou, Yunnan.

#### Host plant.

*Debregeasis* sp.

### ﻿ Gallerucida ornatipennis

Taxon classificationAnimaliaColeopteraChrysomelidae

(Duviver, 1885)

03D0C47A-B27A-5340-A493-405D00639EDA

Fig. 46

Hylaspes? Ornatipennis Duviver, 1885: 397. **TL** China; **TD**ISNB.
Eustetha annulipennis Fairmaire, 1889: 79. Synonymized by Weise 1922: 93.
Eustetha varians Allard, 1891: 233. Synonymized by Kimoto 1989: 238.
Galerucida ornatipennis : Weise 1924: 141.
Galerucida ornatipennis var. decolora Laboissière, 1934: 120. Synonymized by Wilcox 1971: 205.
Galerucida ornatipennis var. violacea Laboissière, 1934: 120. Synonymized by Wilcox 1971: 205.
Galerucida ornatipennis ab. Inornana Mader, 1938: 57. Synonymized by Wilcox 1971: 205.
Galerucida ornatipennis ab. Aeneicollis Mader, 1938: 57. Synonymized by Laboissière 1940: 23 (= var.violacea Laboissière, 1934: 120).
Gallerucida ornatipennis : Gressitt and Kimoto 1963: 728.

#### Other material examined

**(43 spec.).** China: **Guangxi**: 1♂, Guangxi, Linyun Xian, Shali, 28-VIII-1980, unknown, leg.; **Sichuan**: 1♀, Sichuan, Emei Mountain, 10-VI-1955, Ke-Ren Huang & Yin-Tao Jin, leg.; 1♀, Sichuan, Emei Mountain, 760 m, 23-VI-1955, Jin-Hua Li, leg.; 1♀, Sichuan, Emei Mountain, Baoguosi, 550–750 m, 22-VI-1957, You-Cai Lu, leg.; 1♀, Sichuan, Emei Mountain, Qinyinge, 800–1000 m, 22-IV-1957, Ke-Ren Huang, leg.; 1♂, Sichuan, Emei Mountain, 29-VI-1955, Ke-Ren Huang, leg.; 1♂, Sichuan, Emei Mountain, 580–1100 m, 21-VI-1955, Yun-Zhen Zi, leg.; 1♂, Sichuan, Emei Mountain, 580–650 m, 20-VI-1955, Zhong-Lin Ge, leg.; 1♂, Sichuan, Xichang Xian, 1600 m, 24-IX-1960, Xu-Wu Meng, leg.; **Guizhou**: 2♀♀, Guizhou, Guiyang, Huaxi, 1000 m, 3-VII-2006, De-Yan Ge, leg.; **Yunnan**: 1♀, Yunnan, Fengqing Xian, 1500 m, 2-VII-1980, unknown, leg.; 1♀, Yunnan, Jingdong Xian, 1170 m, 28-VI-1956, Krejanovsky, leg.; 1♀, Yunnan, Jingdong Xian, Dongjiafen, 1250 m, 26-VI-1956, Krejanovsky, leg.; 1♀, Yunnan, Jinping Xian, Mengla, 370 m, 22-IV-1956, Ke-Ren Huang et al., leg.; 1♀, Yunnan, Lunan Yizu Zizhixian, Shilin, 1700 m, 9-VII-1956, Krejanovsky, leg.; 1♀, Yunnan, Qiubei Xian, 1320 m, 12-VII-1979, Wen-Zheng Hu, leg.; 1♂, Yunnan, Jingdong Xian, 1170 m, 27-V-1956, Krejanovsky, leg.; 1♂, Yunnan, Jingdong Xian, 1170 m, 27-V-1956, Krejanovsky, leg.; 1♂, Yunnan, Jingdong Xian, 1170 m, 2-VII-1956, Wei Zhang, leg.; 1♂, Yunnan, Jingdong Xian, 1170 m, 7-VI-1956, Wei Zhang, leg.;1♂, Yunnan, Jingdong Xian, Dongjiafen, 1250 m, 30-V-1956, Krejanovsky, leg.; 1♂, Yunnan, Jingdong Xian, Dongjiafen, 1250 m, 6-VI-1956, Wei Zhang, leg.; 1♂, Yunnan, Lu Shui, Laowo, 1670 m, 25-VI-1981, Xue-Zhong Zhang, leg.; 1♂, Yunnan, Lunan Yizu Zizhixian, Shilin, 1700 m, 9-VII-1956, Krejanovsky, leg.; 1♂, Yunnan, Wenshan Xian, 18-VII-1958, unknown, leg.; 2♀♀, Yunnan, Jingdong Xian, 1170 m, 4-VII-1956, Krejanovsky, leg.; 2♀♀, Yunnan, Laowo, 1670 m, 25-VI-1981, Shu-Yong Wang, leg.; 2♂♂, Yunnan, Jingdong Xian, Dongjiafen, 1250 m, 5-VI-1956, Krejanovsky, leg.; 2♂♂, Yunnan, Lunan Yizu Zizhixian, Shilin, 1700 m, 9-VII-1956, Krejanovsky leg.; 3♀♀, 6♂♂, Yunnan, Baoshan Xian to Yongping Xian, 28-V-1955, Tian-Rong Huang, leg. (all IZAS).

#### Distribution.

China: Guangxi, Sichuan, Guizhou, Yunnan.

### ﻿ Gallerucida rubrozonata

Taxon classificationAnimaliaColeopteraChrysomelidae

Fairmaire, 1889

0A7AF2BA-78E7-525C-B398-99AA1B5318D2

Fig. 47

Galerucida rubrozonata Fairmaire, 1889: 75, 79. **TL** China: Sichuan; **TD**MNHN.
Galerucida rubrozonata ab. Atronotata Fairmaire, 1889: 76. Synonymized by Laboissière 1926: 53.
Gallerucida rubrozonata : Gressitt and Kimoto 1963: 731, fig. 188b.

#### Type material.

***Syntype***: labels: 701 // MUS. HIST. NAT. / A. DAVID / Moupin (Thibet), 1871 // *Galerucidarubrozonata* Fairm. // Type // Syntype // Syntype / *Gallerucida* / *rubrozonata* Fairmaire, 1889. (MNHN EC12240).

#### Other material examined

**(7 spec.).** China: **Yunnan**: 1♂, Yunnan, Lijiang, 18-V-1974, leg.; **Sichuan**: 1♀, Sichuan, Emei Mountain, Jiulaodong, 1800–1900 m, 19-VIII-1957, Zong-Yuan Wang, leg.; 1♀, Sichuan, Emei Mountain, Jiulaodong, 1780 m, 18-VIII-1957, You-Cai Lu, leg.; 1♀, Sichuan, Emei Mountain, Xixiangchi, 1800–2000 m, 8-IX-1957, Ke-Ren Huang, leg.; 1♀, Sichuan, Emei Mountain, Jiulaodong, 1800–2000 m, 17-VIII-1957, You-Cai Lu, leg.; 1♀, Sichuan, Emei Mountain, Xixiangchi, 1800–2000 m, 22-V-1957, You-Cai Lu, leg.; 1♀, Sichuan, Emei Mountain, Qinyinge, 800–1000 m, 30-V-1957, You-Cai Lu, leg. (all IZAS).

#### Distribution.

China: Sichuan, Yunnan (new record).

### ﻿ Gallerucida sauteri

Taxon classificationAnimaliaColeopteraChrysomelidae

Chûjô, 1938

8EA60722-8010-5AFB-844D-F85AD2738AFA

Fig. 48

Gallerucida sauteri Chûjô, 1938: 141. **TL** China: Taiwan; **TD**SDEI.
Gallerucida quadraticollis Takizawa, 1978: 127. Synonymized by Kimoto and Chu 1996: 92.

#### Type material.

***Syntypes*.** labels: China, Kankau (Koshun) / Formosa (Taiwan) / H. Sauter V. 1912 // Syntypus // *Galerucida* / *sauteri* / Chûjô/ Det. M. Chûjô // DEI Müncheberg / Col - 09173 / Paralectotypus / *Gallerucidasauteri* / Chûjô, 1938 / des. C. -F. Lee, 2017. (SDEI #303336).; China, Koshun / Formosa (Taiwan) / H. Sauter / VIII. 18 // *Galerucida* / *sauteri* / Chûjô / Det. M. Chûjô // DEI Müncheberg / Col – 09172 // Paralectotypus / *Gallerucidasauteri* / Chûjô, 1938 / des. C. -F. Lee, 2017. (SDEI #303335).

#### Distribution.

China: Taiwan.

#### Host plant.

*Tetrastigmaformosanum*.

### ﻿ Gallerucida tenuefasciata

Taxon classificationAnimaliaColeopteraChrysomelidae

Fairmaire, 1888

A088F3F9-E75B-52EB-9D90-865ED73786D4

Fig. 49

Galerucida tenuefasciata Fairmaire, 1888: 40. **TL** China: Yunnan; **TD**MNHN.
Galerucida potanini Ogloblin, 1936: 358, 442, 445, fig. Synonymized by Gressitt and Kimoto 1963: 734.
Gallerucida tenuefasciata : Gressitt and Kimoto 1963: 734, fig. 188c.

#### Type material.

***Syntype***: labels: China, Yunnan // *Galerucida* / *tenuefasciata* / Fairm. // Ex-Musaeo / L. Fairmaire / 1893. (MNHN).

#### Distribution.

China: Sichuan, Yunnan.

### ﻿ Gallerucida tricolor

Taxon classificationAnimaliaColeopteraChrysomelidae

Gressitt & Kimoto, 1963

E401F0F8-1FC9-5CDE-A4D7-ECC462EAFB34

Fig. 50

Gallerucida tricolor Gressitt & Kimoto, 1963: 736, fig. **TL** China: Yunnan; **TD**NHMB.

#### Type material.

***holotype***: labels: China, prov. Yunnan. / Vallis flumin. / Soling-ho. // Holotype ♂ / *Gallerucida* / *tricolor* / Gressitt & Kimoto // Museum Frey / Tutzing // *Galerucida* / sp. nov. 2 / *tricolor* Hole 161 / Det. S. Kimoto. (NHMB)

#### Other material examined.

China: **Yunnan**: 1♂, Yunnan, Liusheng, Liude, 2400 m, 18-VIII-1984, Shu-Yong Wang, leg. (IZAS).

#### Distribution.

China: Yunnan.

##### ﻿Description of new species

### ﻿ Gallerucida fortispina

Taxon classificationAnimaliaColeopteraChrysomelidae

Xu & Yang sp. nov.

E22EA9C7-20B3-52FB-B7E5-252B36F4A0B4

https://zoobank.org/19F245F1-6963-4917-BDEF-A70E0AA8FE26

Figs 1–7

#### Type material.

***Holotype*.** China: ♂, Guangxi, Nape, Nongxin / 1000 m / 12-IV-1998, Chao-Dong Zhu, leg. (IZAS).

***Paratypes*.** China: 1♂, Guangxi, Nape, Beidou / 550 m / 12- IV-1998, Chun-Sheng Wu, leg.; 1♂, Guangxi, Nape, Beidou / 550 m / 10-IV-1998, Chao-Dong Zhu, leg.; 1♂, Guangxi, Nape, Beidou / 550 m / 10-IV-1998, Tian-Shan Li, leg. (all IZAS).

#### Description.

Length 7.5–8.0 mm, width 4.0–5.0 mm (*n* = 4, including holotype). Holotype length 7.5 mm, width 4.0 mm

**Figures 1–7.**
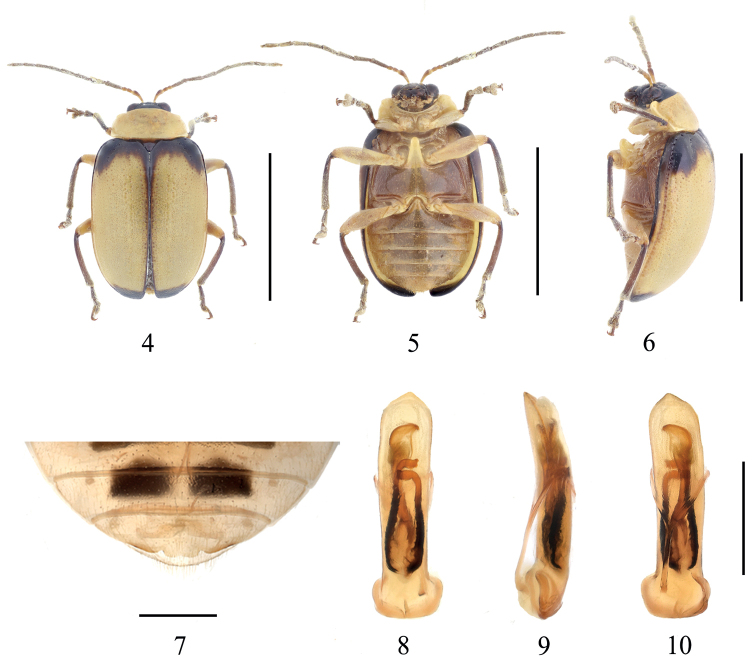
*Gallerucidafortispina* sp. nov. **1** dorsal view (paratype) **2** ventral view (paratype) **3** lateral view (paratype) **4** ventral view of 5^th^ ventrite, male (holotype) **5** aedeagus, dorsal view (holotype) **6** ditto, lateral view (holotype) **7** ditto, ventral view (holotype). Scale bars: 5 mm (**1–3**); 1 mm (**4–7**).

**Male**: Body oval. General color (Figs 1–3) light yellow; first three segments of antennae yellowish brown, rest brown; head and scutellum black; basal area and apical area of elytra with black bands, epipleura and suture dark brown; meso- and meta- sternum, abdomen brown except apical of anterior metasternal process light yellow; femora light yellow, tibiae and tarsi brown.

Head distinctly narrower than prothorax; occiput slightly convex, with sparsely punctures; frontoclypeus surface smooth; frontal tubercle developed, triangular. Antennae longer than 1/2 length of elytra, basal three segments moderately shiny, from 4^th^ segment, covered with fine pale hairs; length ratios of antennomeres I–V 1.0: 0.3: 0.3: 1.1: 0.9, in length 3^rd^ and 2^nd^ segment subequal, 4^th^ segment longest, 5^th^–11^th^ segment subequal. Pronotum transverse, ~ 2.5 × as broad as long; lateral margin subparallel, anterior margin concave, anterior corner indistinct, basal margin convex; disc area impunctate, middle area with a transverse shallow depression. Anterior metasternal process extending obviously beyond the front edge of the meso-coxal cavities, surface smooth. Scutellum triangular, smooth and impunctate. Elytra 1.5 × as long as broad, lateral margin after half evenly narrowing; disc slightly convex, with fine regular punctures, space between punctures larger than the diameter of puncture; epipleura surface smooth. Last sternite of male with distinct trilobite concavities, middle lobate wave-like (Fig. 4).

Aedeagus almost parallel-sided from base to apex in dorsal view, apex obtuse angled, slightly curved towards ventral side; internal sclerites strong (Figs 5–7), median longitudinal sclerite almost reaching apex of aedeagus, with apex enlarged; a pair of lateral longitudinal sclerites, converging apically.

#### Derivatio nominis.

The specific epithet *fortispina* is formed from the Latin adjective *fortis* (strong) and the Latin noun *spina* (spine); it refers to the endophallic sclerite complex.

#### Distribution.

China: Guangxi.

#### Diagnosis.

*Gallerucidafortispina* closely resembles *G.sauteri* Chûjô, but the former has a larger body size, black head, light yellow impunctate pronotum, irregularly punctate elytra, light yellow femora, and metasternal process exceeding mesosternum.

### ﻿ Gallerucida levifasciata

Taxon classificationAnimaliaColeopteraChrysomelidae

Xu & Nie sp. nov.

D632A258-60E3-5A77-97F9-52F5D0CA3811

https://zoobank.org/8835C446-57D1-4A27-99F9-6DE40573E9D6

Figs 8–14

#### Type material.

***Holotype*.** China: ♂, Gansu, Gannan / 1992 // unknown, leg. (IZAS).

***Paratype*.** China: 1♂, Gansu, Gannan / 1992 // unknown, leg. (IZAS).

#### Description.

Length 5.5–6.0 mm, width 3.0–3.5 mm (*n* = 2, including holotype). Holotype length 5.5 mm, width 3.0 mm.

**Figures 8–14:**
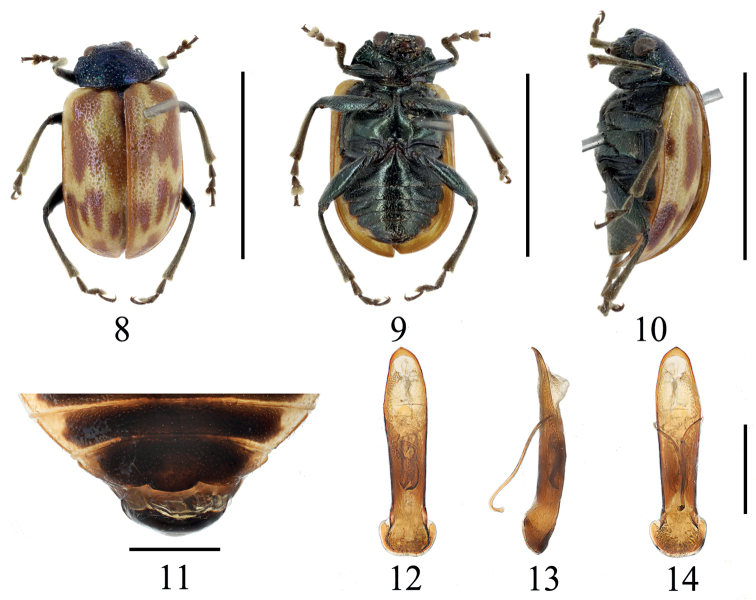
*Gallerucidalevifasciata* sp nov (holotype) **8** dorsal view **9** ventral view **10** lateral view **11** ventral view of 5^th^ ventrite, male **12** aedeagus, dorsal view **13** ditto, lateral view **14** ditto, ventral view. Scale bars: 5 mm (**8–10**); 1 mm (**11–14**).

**Male**: Body oval and colorful (Figs 8–10). First three segments of antennae light reddish brown, 4^th^ segment brown, remaining segments lost; head, pronotum and scutellum atropurpureus with metallic luster; elytra and epipleura yellowish brown, elytra with an irregular brown band from scutellum to middle area, and five brown spots after middle distributed in two rows, first row arranged four slight slim brown strips, second row arranged one round brown spot reaching elytron apical area; suture brown; body ventral surface and femora dark green; apical area of anterior metasternal process, tibiae and tarsi dark brown.

Head distinctly narrower than prothorax; occiput concave, with strong sparsely punctures; frontoclypeus triangular, slightly convex, with punctures; frontal tubercle developed. Antennae (only basal four segments remain) basal three segments moderately shiny, 4^th^ segment covered with fine pale hairs; length ratios of segments I–IV 1.0: 0.3: 0.3: 1.7. Pronotum transverse, ~ 2.3 × as broad as long, lateral margin rounded, anterior margin concave, anterior corner indistinct, basal margin convex, disc area with densely strong punctures. Anterior metasternal process reach apex of the mesocoxal cavities, with punctures. Scutellum triangular, slightly rounded apically, with sparsely punctures. Elytra subparallel-sided, 1.6 × as long as broad; disc slightly convex, two types of irregular punctures in elytra: space between smaller punctures smaller than diameter of puncture, epipleura surface smooth. Last sternite of male with distinctly light trilobite concavities (Fig. 11).

Aedeagus slight broadened in middle area in dorsal view, apex obtuse angled, slightly curved towards ventral side; internal sclerites (Figs 12–14): a pair of lateral sclerites shorter and converging in apical area in dorsal view.

#### Derivatio nominis.

The specific epithet *levifasciata* is formed from the Latin adjectives *levis* (meaning light) and *fasciata* (banded); named for the irregular light brown band on the elytra adjacent to the scutellum.

#### Distribution.

China: Gansu.

#### Diagnosis.

*Gallerucidalevifasciata* closely resembles *G.bifasciata* Motschulsky, but it can be distinguished from the latter by: ventral surface color, scutellum with punctures and elytra with two types of large and small irregular punctures; *G.levifasciata* is also similar to *G.piceusfasciata* sp. nov., but the latter has a yellow metasternal process apically and basal abdominal sternite.

### ﻿ Gallerucida nigrovittata

Taxon classificationAnimaliaColeopteraChrysomelidae

Xu & Yang sp. nov.

F3E5BA58-9F2A-5BB3-98D5-DD2B43AEE3BC

https://zoobank.org/7823DBE0-689D-4441-981D-A7891E8B21C2

Figs 15–21

#### Type material.

***Holotype*.** China: ♂, Yunnan, Jinping, He-tou-zhai, 2000 m / 10-V-1956 / Ke-Ren Huang et al., leg. (IZAS).

***Paratype*.** China: 3♂♂, Yunnan, Jinping, He-tou-zhai, 2000 m / 10-V-1956 / Ke-Ren Huang et al., leg. (all IZAS).

#### Description.

Length 5.0–5.5 mm, width 3.0–3.5 mm. (*n* = 4, including holotype). Holotype length 5.3 mm, width 3.3 mm.

**Figures 15–21.**
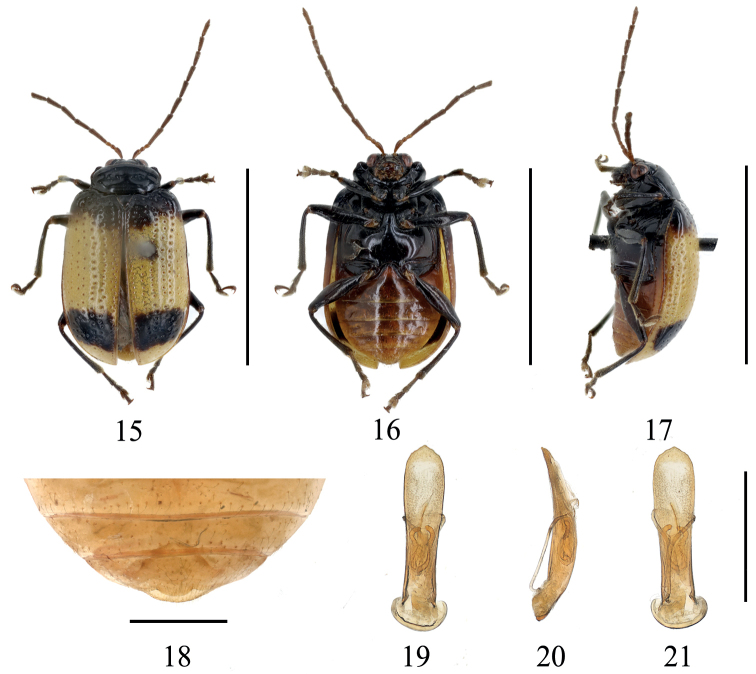
*Gallerucidanigrovittata* sp. nov. **15** dorsal view (holotype) **16** ventral view (holotype) **17** lateral view (holotype) **18** ventral view of 5^th^ ventrite, male (paratype) **19** aedeagus, dorsal view (paratype) **20** ditto, lateral view (paratype) **21** ditto, ventral view (paratype). Scale bars: 5 mm (**15–17**); 1 mm (**18–21**).

**Male**: Body oval. General color (Figs 15–17) black; antennae brown; elytra yellow, basal and subapical area of each elytron with transverse black stripe; 1/3 of epipleura black, rest yellow-brown; meso- and meta- sternum brown or black; ventral surface of abdomen reddish brown; tibiae and tarsi brown or black.

Head narrower than prothorax; occiput distinctly concave, with sparse faint punctures; frontoclypeus surface smooth; frontal tubercle developed, nearly square. Antennae longer than 1/2 length of elytron, basal three segments moderately shiny, from 4^th^ segment, covered with fine pale hairs; length ratios of antennomeres I–V, 1.0: 0.5: 0.5: 2.0: 1.6, 3^rd^ segment and 2^nd^ segment subequal, 4^th^ segment longest, 5^th^-11^th^ segments subequal. Pronotum transverse, ~ 2.2 × as broad as long, lateral margin rounded, after middle slight narrow, anterior margin concave, anterior corner distinct, basal margin convex, disc area with a pair of distinct transverse depressions with strong punctures;anterior metasternal process reaching apex of mesocoxal cavities, surface smooth. Scutellum triangular, impunctate. Elytra subparallel-sided, 1.4 × as long as broad, disc slightly convex, with regular punctures, space between punctures larger than diameter of puncture, moderately punctured in rows, ~ 14 rows across central portion. Last sternite of male with slight trilobite concavities (Fig. 18).

Aedeagus slightly broadened in the apical area of dorsal view, apex forming distinct obtuse angle, curved slightly towards ventral side; internal sclerites (Figs 19–21): median sclerite longitudinal, slim in apex, reaching to 2/3 length of aedeagus, a pair of lateral longitudinal sclerites, convergent apically.

#### Derivatio nominis.

The specific epithet *nigrovittata*, is formed from the Latin adjective *niger* (black) and the Latin adjective *vittata* (banded) referring to the transverse subapical black stripe on the elytron.

#### Distribution.

China: Yunnan.

#### Diagnosis.

*Gallerucidanigrovittata* closely resembles *G.sauteri* Chûjô, but the former differs in having a black pronotum, brown or blackish brown antennae, brown or black meso- and meta- sternum and reddish brown abdomen.

### ﻿ Gallerucida octodecimpunctata

Taxon classificationAnimaliaColeopteraChrysomelidae

Xu & Yang sp. nov.

BED211D9-A556-5C58-964D-4E01DD3A343F

https://zoobank.org/651A3EAF-B572-49B0-9E12-E7DC4DC15681

Figs 22–28

#### Type material.

***Holotype*.** China: ♂, Yunnan, Xishuangbanna, Menglun / 550 m // 15-VII-1959 / Fa-Cai Zhang, leg. (IZAS).

***Paratype*.** China: 1♂, Yunnan, Yiwuzhen / 650 m // 26-VII-1956 / Fa-Cai Zhang leg. (IZAS)

#### Description.

***Holotype*.** Length 7.0 mm, width 4.0 mm.

**Figures 22–28.**
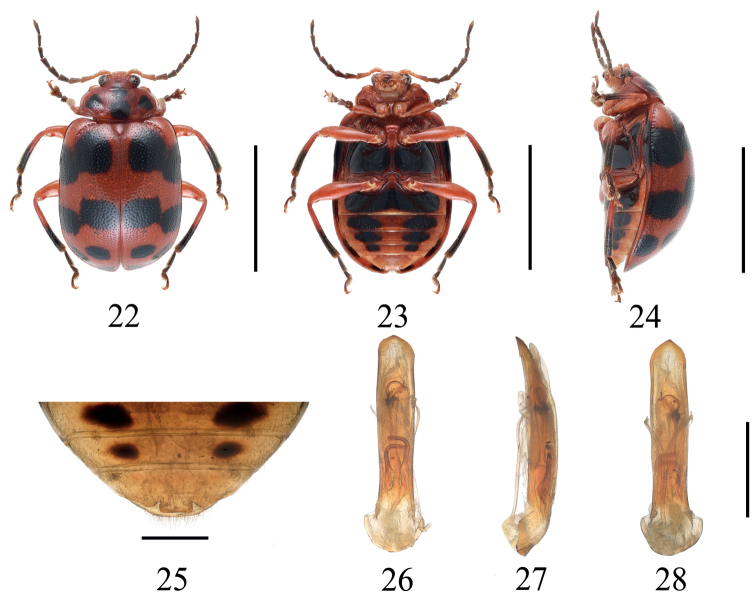
*Gallerucidaoctodecimpunctata* sp. nov. **22** dorsal view (paratype) **23** ventral view (paratype) **24** lateral view (paratype) **25** ventral view of 5^th^ ventrite, male (paratype) **26** aedeagus, dorsal view (holotype) **27** ditto, lateral view (holotype) **28** ditto, ventral view (holotype). Scale bars: 5 mm (**22–24**); 1 mm (**25–28**).

**Male**: Body oval. General color (Figs 22–24) red; basal four segments of antennae orange, the rest brown; occiput with a black spot near anterior margin of pronotum; pronotum with four black spots: middle area of disc with two adjacent longitudinal black spots, each side with one round black spot, respectively; elytra with seven rounded black spots, distributed in four rows, arrangement for 2: 2: 2: 1, last one situated at apical area; middle area of epipleura black, rest red; meso - meta sternum black, anterior metasternal process dark red, ventral surface of each visible abdomen except the last one with two black spots; 1/2 of tibiae and tarsi black.

Head distinctly narrower than prothorax, occiput concave, with faintly punctures; frontoclypeus surface smooth; frontal tubercle developed, square. Antennae shorter than 1/2 length of elytra, basal three segments moderately shiny, from 4^th^ segment, covered with short pale hairs; length ratios of antennomeres I–V, 1.0: 0.4: 0.3: 1.2: 1.1, 3^rd^ segment slightly shorter than 2^nd^ segment in length, 5^th^ -11^th^ segments subequal, the last segment longest. Pronotum transverse, ~ 2.4 × as broad as long, lateral margin rounded, anterior margin concave, anterior corner indistinct, basal margin convex, disc area with uniformly distributed fine punctures, near lateral margin with shallow inclined depression. Anterior metasternal process reaching apex of mesocoxal cavities, surface smooth, apex square. Scutellum triangular, impunctate. Elytra subparallel-sided, 1.6 × as long as broad, disc slightly convex, disc with uniformly distributed fine punctures, space between punctures larger than the diameter of puncture. Last sternite of male with distinctly trilobite concavities (Fig. 25).

Aedeagus almost parallel-sided from base to apex in dorsal view, apex obtusely angled, slightly curved towards ventral side; internal sclerites (Figs 26–28): median sclerite longitudinal, extending almost to apex of aedeagus, with apex enlarged; a pair of lateral longitudinal sclerites, ½ length of median sclerite, convergent apically.

#### Derivatio nominis.

The specific epithet *octodecimpunctata*, comes from the Latin numeral, *octodecim*- (eighteen) and Latin adjective *punctata* (spotted); it refers to eighteen black spots on the pronotum (4) and elytra (14).

#### Distribution.

China: Guangxi.

#### Diagnosis.

This species is similar to *Gallerucidabalyi* (Duvivier), but *G.octodecimpunctata* has brown antennae except the basal segments orange, red femora and black distal 1/2 of tibiae, while *G.balyi* has antennae total yellowish brown and femora and tibiae yellow.

### ﻿ Gallerucida piceusfasciata

Taxon classificationAnimaliaColeopteraChrysomelidae

Xu & Yang sp. nov.

3D806DC9-0516-53D7-B01D-8C31F3360C1B

https://zoobank.org/B4EB5AC0-AC8F-41EA-B6BF-C339D4AD58D4

Figs 29–35

#### Type material.

***Holotype*.** China: ♂, Sichuan, Badan / 19-X-1979 // unknown, leg. (IZAS).

***Paratype*.** China: ♀, Sichuan, Badan / 19-X-1979 // unknown, leg. (IZAS; destroyed).

#### Description.

Length 6.5–6.8 mm, width 3.8 mm (*n* = 2, including holotype). Holotype length 6.8 mm, width 3.8 mm

**Figures 29–35.**
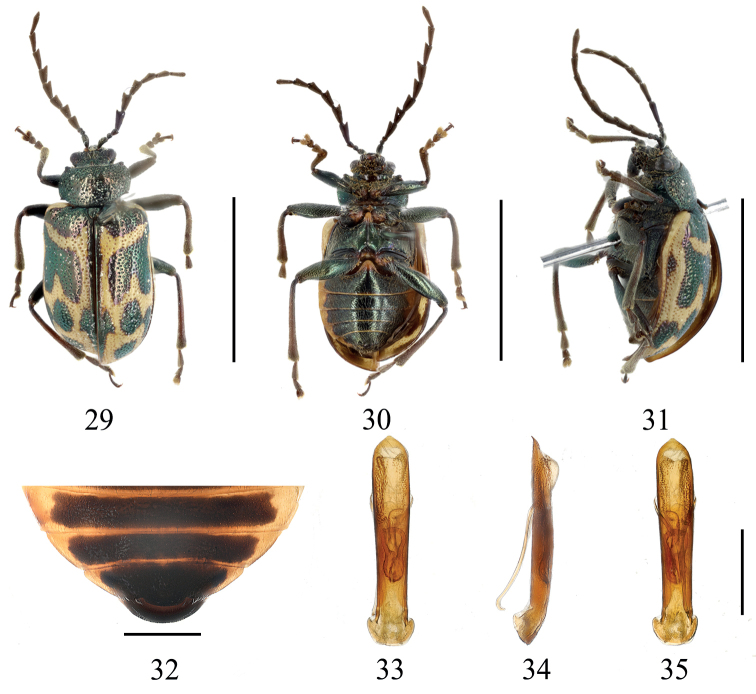
*Gallerucidapiceusfasciata* sp. nov. (holotype) **29** dorsal view **30** ventral view **31** lateral view **32** ventral view of 5^th^ ventrite, male **33** aedeagus, dorsal view **34** ditto, lateral view **35** ditto, ventral view. Scale bars: 5 mm (**29–31**); 1 mm (**32–35**).

**Male**: Body oval. General color (Figs 29–31) generally dark green with metallic luster; first three segments of antennae dark green, rest brown; elytra with four pairs of yellow stripes, one pair at elytron humerus, second pair transverse stripe with vertical extension located in 1/3 elytron, near suture, third pair at middle area forming irregular waved stripes, last pair subapical forming a circle; epipleuron yellow; apical of anterior metasternal process and first visible abdomen sternite yellowish brown; tibiae and tarsi brown.

Head distinctly narrower than prothorax, occiput and frontoclypeus with strong punctures and distinct hairs, frontal tubercle developed. Antennae reaching 1/2 length of elytron, basal three segments with long hairs, remaining segments covered with short pale hairs; length ratios of antennomeres I–V, 1.0: 0.5: 0.5: 1.6: 1.5, 3^rd^ and 2^nd^ segment subequal in length, length from 4^th^ segment decreased gradually. Pronotum transverse, ~ 1.8 × as broad as long, lateral margin rounded, anterior margin concave with dense setae, anterior corner distinct, basal margin convex, posterior corner distinct, disc area with densely strong punctures. Anterior metasternal process not reaching apex of mesocoxal cavities, surface smooth, sides with hairs. Scutellum triangular, rounded apically, with faint punctures. Elytra subparallel-sided, 1.4 × as long as broad, disc slightly convex, with irregular punctures in two sizes, space between smaller punctures little larger the diameter of punctures, space between larger punctures smaller the diameter of punctures. Last sternite of male with shallow trilobite concavities (Fig. 32).

Aedeagus almost parallel-sided from base to apex in dorsal view, apex obtuse angled, slightly curved towards ventral side; internal sclerites (Figs 33–35): median sclerite longitudinal, slight longer than lateral sclerites in length, apically bending; a pair of lateral sclerites close to each other at apical area.

#### Derivatio nominis.

The specific epithet *piceusfasciata*, is formed from the Latin adjectives, *piceus* (dark green) and *fasciata* (banded); referring to the elytra being general dark green with yellow bands.

#### Distribution.

China: Sichuan.

#### Diagnosis.

This species is similar to *Gallerucidabifasciata* Motschulsky. The main differences are the following: *G.piceusfasciata* has subequal 2^nd^ and 3^rd^ antennal segments, sparsely punctured scutellum, and two types of large and small irregular punctures on the elytra.

### ﻿ Gallerucida rufipectoralis

Taxon classificationAnimaliaColeopteraChrysomelidae

Xu & Nie sp. nov.

36AC1961-417F-5651-B2B4-933D6ADA2DD6

https://zoobank.org/59B59699-ABBD-4DE1-B5E2-F1524C4131CA

Figs 36–42

#### Type material.

***Holotype*.** China: ♂, Yunnan, Lushui / Laochuang, 2430 m // 19-VI-1981 / Shu-Yong Wang, leg. (IZAS).

***Paratypes*.** China: 3♂♂, Yunnan, Lushui / Laochuang, 2430 m // 19-VI-1981 / Shu-Yong Wang, leg.; 1♂, Yunnan, Yunlongxian / Zhibenshan, 2250 m // 21-VI-1981 / Shu-Yong Wang, leg.; 1♂, Yunnan, Yunlongxian / Zhibenshan, 2550 m // 22-VI-1981 / Shu-Yong Wang, leg.; 1♂, Yunnan, Yunlongxian / Zhibenshan, 2550 m // 22-VI-1981 / Su-Bai Liao, leg. (all IZAS).

#### Description.

Length 5.0–5.5 mm, width 3.0–4.0 mm (*n* = 7, including holotype). Holotype length 5.3 mm, width 3.1 mm.

**Figures 36–42.**
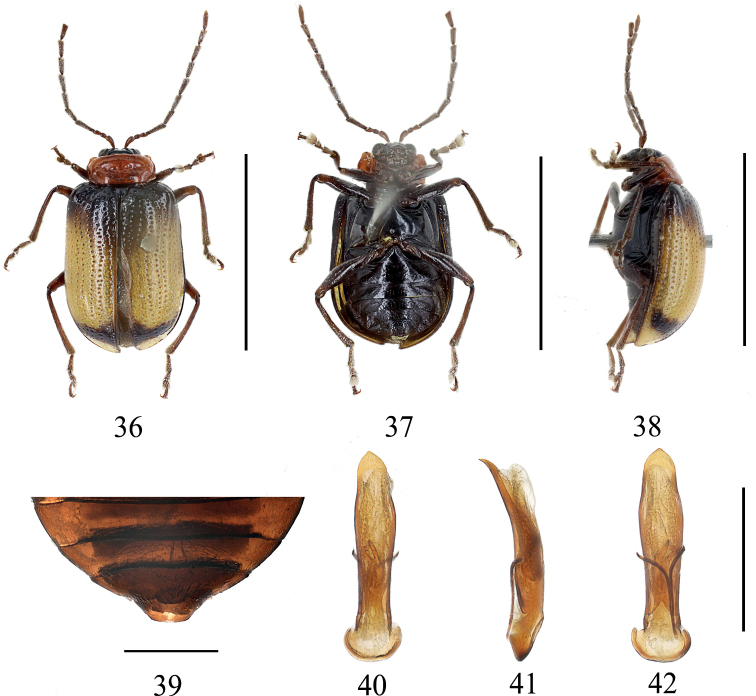
*Gallerucidarufipectoralis* sp. nov. **36** dorsal view (holotype) **37** ventral view (holotype) **38** lateral view (holotype) **39** ventral view of 5^th^ ventrite, male (paratype) **40** aedeagus, dorsal view (paratype) **41** ditto, lateral view (paratype) **42** ditto, ventral view (paratype). Scale bars: 5 mm (**36–38**); 1 mm (**39–42**).

**Male**: Body oval. General color (Figs 36–38) black; the first three antennal segments light reddish brown, rest brown; pronotum reddish brown; elytra yellowish brown, basal area of elytra with a gradually blurred black band, subapically with a transverse black stripe; epipleura black; femora dark brown, tibiae and tarsi brown.

Head distinctly narrower than prothorax, occiput sparsely punctures, with a transverse depression; frontoclypeus slightly convex, triangular, with sparsely punctures; frontal tubercle developed, rounded. Antennae longer than 1/2 length of elytron, basal three segments moderately shiny, from 4^th^ segment, covered with fine pale hairs; length ratios of antennomeres I–V, 1.0: 0.5: 0.5: 1.6: 1.2, 3^rd^ and 2^nd^ segment subequal in length, 4^th^ segment longest, 5^th^ -11^th^ segments subequal. Pronotum transverse, approximately twice as broad as long, lateral margin subparallel, anterior margin concave, anterior corner indistinct, basal margin convex, disc area with sparse strong punctures and two shallow transverse depressions. Anterior metasternal process not reaching apex of mesocoxal cavities, surface with punctures. Scutellum triangular, surface smooth. Elytra subparallel-sided, 1.6 × as long as broad, disc slightly convex, with regular punctures in nearly 18 rows, space between punctures larger than the diameter of puncture. Last sternite of male with shallow trilobite concavities (Fig. 39).

Aedeagus expanded at 1/2 and 2/3 distance between base to apex, apex sharp in dorsal view, distinctly curved towards ventral side; internal sclerites (Figs 40–42), a pair of sclerites bending apically.

#### Derivatio nominis.

The specific epithet *rufipectoralis*, is formed from the Latin adjectives *rufus* (red) and *pectoralis* (thorax) referring to the pronotum reddish brown.

#### Distribution.

China: Yunnan.

#### Diagnosis.

*Gallerucidarufipectoralis* closely resembles *G.sauteri* Chûjô, but the former has dark brown femora, black head and ventral surface, subequal 3^rd^ and 2^nd^ segments of the antennae, and punctured pronotum depression.

### ﻿ Aplosonyx gansuica

Taxon classificationAnimaliaColeopteraChrysomelidae

(Chen, 1942) comb. nov.

1EDFFD92-20EF-5D0B-AC7C-D02B61C7DDB1

Fig. 51

Galerucida gansuica Chen, 1942: 38. **TL** China: Gansu; **TD**IZAS.
Gallerucida gansuica : Gressitt and Kimoto 1963: 724, fig. 188a.

#### Type material.

***Holotype***: labels: China, Gansu / 18-V-1919 // Holotype // IOZ 215680 // *Galerucidagansuica* / Chen S-H. (IZAS).

#### Distribution.

China: Gansu, Hubei, Sichuan, Guizhou.

**Figures 43–51.**
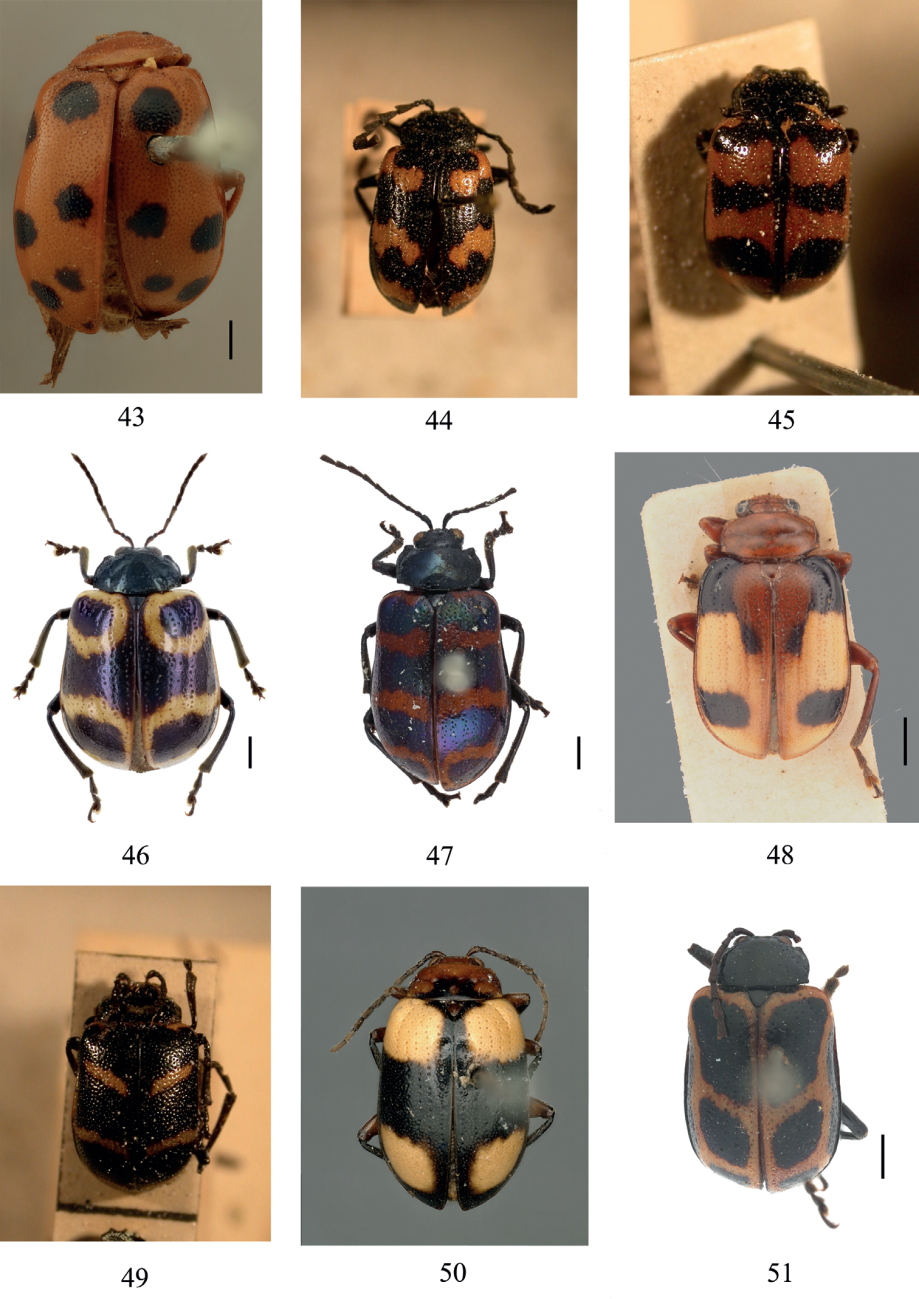
Habitus. **43***G.balyi* (Paratype, ISNB) **44***G.bifasciata* (Syntype, MNHN) **45***G.nigropicta* (Syntype, MNHN) **46***G.ornatipennis* (identified species) **47***G.rubrozonata* (Syntype, MNHN) **48***G.sauteri* (Syntype, SDEI) **49***G.tenuefasciata* (Syntype, MNHN) **50***G.tricolor* (Holotype, NHMB) **51***Aplosonyxgansuica* (Holotype, IZAS). Scale bars: 1 mm.

## Supplementary Material

XML Treatment for Gallerucida

XML Treatment for Gallerucida balyi

XML Treatment for Gallerucida bifasciata

XML Treatment for Gallerucida nigropicta

XML Treatment for Gallerucida ornatipennis

XML Treatment for Gallerucida rubrozonata

XML Treatment for Gallerucida sauteri

XML Treatment for Gallerucida tenuefasciata

XML Treatment for Gallerucida tricolor

XML Treatment for Gallerucida fortispina

XML Treatment for Gallerucida levifasciata

XML Treatment for Gallerucida nigrovittata

XML Treatment for Gallerucida octodecimpunctata

XML Treatment for Gallerucida piceusfasciata

XML Treatment for Gallerucida rufipectoralis

XML Treatment for Aplosonyx gansuica
